# Application of global trigger tool to determine the prevalence of adverse drug reactions in adult patients admitted to general and COVID-19 intensive care units

**DOI:** 10.3389/fphar.2025.1514942

**Published:** 2025-05-20

**Authors:** Rafael Nogueira de Souza, Marília Berlofa Visacri, Fabiana Bragança Albanese Trotta, Deise de Souza Ventura, Mauricio Wesley Perroud, Patrícia Moriel

**Affiliations:** ^1^ School of Medical Sciences (FCM), Universidade Estadual de Campinas (UNICAMP), Campinas, São Paulo, Brazil; ^2^ Faculty of Pharmaceutical Sciences (FCF), Universidade de São Paulo (USP), Sao Paulo, Brazil; ^3^ Hospital Estadual de Sumaré Dr. Leandro Francheschini (HES), Sumaré, São Paulo, Brazil; ^4^ Faculty of Pharmaceutical Sciences (FCF), Universidade Estadual de Campinas (UNICAMP), Campinas, São Paulo, Brazil

**Keywords:** pharmacovigilance, intensive care unit, adverse drug reaction, trigger tool, COVID-19

## Abstract

**Objective:**

The primary aim of this study was to determine the prevalence of adverse drug reactions (ADRs) in adult patients admitted to a general adult intensive care unit (G-ICU) and a COVID-19 adult intensive care unit (C19-ICU). The secondary aims were to characterize patients in both ICUs; identify factors associated with the occurrence of ADRs; assess the performance of triggers in detecting ADRs; describe ADRs in terms of severity, mechanism, causality, and suspected drugs; and compare the trigger tool methodology with spontaneous reporting.

**Methods:**

This was a descriptive and retrospective study involving the application of triggers adapted from the Global Trigger Tool to identify ADRs through the analysis of physical and electronic medical records, medical prescriptions, and laboratory test results of adult patients admitted to the G-ICU and C19-ICU of a tertiary hospital in Sumaré (HES), São Paulo, Brazil, from January 2020 to December 2020. The patients were characterized by sex, age, length of stay, clinical outcome (discharge or death), and sequential organ failure assessment (SOFA) scores. The performance of triggers in detecting ADRs was determined by calculating positive predictive value (PPV). ADRs were characterized by severity, mechanism, causality, and suspected drugs. The 2020 spontaneous reporting database at the HES was analyzed, and ADRs from the ICUs were identified.

**Results:**

The study evaluated 135 patients (56.3% from the G-ICU and 43.7% from the C19-ICU), with a predominance of males (54.8%) and a mean age of 61.0 ± 15.1 years. The mean hospital stay was 13.0 ± 11.0 days, the average SOFA score throughout hospitalization was 8.4 ± 3.8, and the ICU mortality rate was 69.6%. Of the 135 admitted patients, 55 (40.7%) presented with at least one ADR, of which 31 (52.5%) were admitted to the C19-ICU. The length of hospitalization was associated with the presence of ADR in both ICUs studied and age only in the C19-ICU. Additionally, patients admitted to the C19-ICU had a 2.4 times higher risk of developing ADRs. A total of 85 ADRs were identified, 65 (76.5%) of which occurred through triggers. The triggers with the best performance in detecting ADRs, with a PPV of 100%, were “Partial Thromboplastin Time >50,” “Skin Rash,” “Protamine,” and “Hydroxyzine.” Most ADRs were moderate (56.5%), Type A (96.5%), and classified as possible (64.7%). Insulin was the drug most frequently associated with ADRs, with 22 occurrences. Only five ADRs in ICU patients were spontaneously reported in 2020.

**Conclusion:**

Of all the patients, 40.7% experienced at least one ADR during hospitalization. The number of ADRs identified by the trigger tool was significantly higher than those reported spontaneously. This demonstrates that using triggers to investigate ADRs is an effective method to significantly enhance an institution’s pharmacovigilance actions.

## 1 Introduction

The World Health Organization (WHO) (World Health Organization, 2023) defines pharmacovigilance as science and activities related to the detection, evaluation, understanding, and prevention of adverse events related to the use of medicines (adverse drug events, ADEs), such as adverse drug reactions (ADRs). Pharmacovigilance plays a fundamental role in ensuring the safety and effectiveness of medicines after they have been marketed (World Health Organization, 2009).

According to the WHO, an ADR is defined as any harmful effect caused by a drug when used in doses typically administered to humans for the prevention, diagnosis, or treatment of a disease or physiological alteration (Gupta et al., 2015). To effectively manage these reactions, it is crucial to apply defined and structured methods (Piccirillo and Parish, 2022).

Pharmacovigilance uses various methods to identify, evaluate, and understand the safety of medicines. These methods are generally divided into two categories: passive surveillance, which primarily relies on spontaneous reporting, and active surveillance, which involves a systematic search for ADRs using retrospective triggers in medical records (Chatterjee and Aparasu, 2023). One widely used active surveillance method is the Global Trigger Tool (GTT), which is based on an adaptation of the medical record review methodology developed by Harvard University (Leape et al., 1991).

Although spontaneous reporting is the most widely used method worldwide, studies have shown that the ADR notification rates are low, ranging from 9% to 15% (Lazarou et al., 1998; Chan et al., 2016; Impicciatore et al., 2001; Prashanthi et al., 2019). Several factors contribute to underreporting in spontaneous notification systems, such as overburdened health professionals and a lack of appreciation for this type of reporting (García-Abeijon et al., 2023). A systematic review by Varallo et al. (2014) identified ignorance, insecurity, and indifference towards the patients as the main causes of underreporting by health professionals.

Critically ill patients in intensive care units (ICUs) are more susceptible to ADRs owing to various factors, reflecting the clinical complexities and fragility of these patients. These patients are often subjected to polypharmacy and non-physiological conditions, generating a state of stress that can alter the body’s response to drugs (Kane-Gill and Devlin, 2006). In ICUs, ADRs are of extreme importance because of not only clinical factors but also their impact on hospitalization duration. A previous study indicated that ICU stay is prolonged in patients with ADRs, which leads to an increase in hospitalization costs (Sendekie et al., 2023).

Therefore, seeking new reporting strategies and other pharmacovigilance actions is essential to ensure the safe use of medicines and the quality of care provided to these patients (Trifirò and Crisafulli, 2022). The primary objective of this study was to use an adapted GTT to determine the prevalence of ADRs in critically ill patients admitted to two adult ICUs: one general ICU and the other dedicated to COVID-19 patients at a general university hospital in the state of São Paulo, Brazil. The secondary aims were to characterize patients in both ICUs; identify factors associated with the occurrence of ADRs; assess the performance of triggers in detecting ADRs; describe ADRs in terms of severity, mechanism, causality, and suspected drugs; and compare the trigger tool methodology with spontaneous reporting.

## 2 Materials and methods

### 2.1 Study design

This descriptive and retrospective study involved the analysis of physical and electronic medical records, medical prescriptions, and laboratory test results of adult patients admitted to the general adult ICU (G-ICU) and COVID-19 adult ICU (C19-ICU) at the State Hospital of Sumaré (HES) from 1 January 2020 to 31 December 2020, who met the inclusion criteria listed below.

### 2.2 Eligibility criteria

The inclusion criteria for the study were as follows: adult patients (>18 years) of both sexes who were admitted to the G-ICU and C19-ICU for more than 24 h and had at least one prescribed medication. Patients with incomplete medical records (i.e., those lacking at least one clinical note from a doctor or nurse) were excluded.

### 2.3 Demographic and clinical data

The patients were characterized by sex (male or female), age (years), length of stay (days), and place of hospitalization: G-ICU or C19-ICU. The clinical outcomes of patients (death or discharge) were determined based on their medical progress. Sequential organ failure assessment (SOFA) scores (Vincent et al., 1996), reflecting the function of various organs and systems (respiratory, cardiovascular, renal, neurological, hepatic, and hematological), were collected according to the medical classifications recorded in the patient records. The SOFA score was calculated by assigning points (0–4; 0 = normal, 4 = severe dysfunction) to each of the following variables: the ratio of arterial oxygen tension (PaO_2_) to the fraction of inspired oxygen (FiO_2_), Glasgow Coma Scale score, mean arterial pressure, serum creatinine level, bilirubin level, and platelet count. The total SOFA score was obtained by summing the points for all variables, ranging from 0 to 24. These scores were documented at least every 48 h. The values presented in this study included the score upon patient’s admission to the ICU, the average score for each patient throughout their ICU stay, and the minimum and maximum scores for each patient throughout their ICU stay. Additionally, the timing of ADR occurrence during hospitalization was assessed.

### 2.4 GTT adaptation

The triggers were adapted from the original GTT methodology (Institute for Healthcare Improvement, 2009) to align with the characteristics of the ICUs at the hospital where the research was conducted. Medication and care modules were utilized, with some modifications. In the medication module, *Clostridium difficile Positive Stool* was excluded owing to the suspension of stool assays during the study period. *Partial Thromboplastin Time (PTT) Greater than 100 Seconds* was adjusted to greater than 50 s, as the ICUs in this study considered this value abnormal. *International Normalized Ratio (INR) Greater than 6* was adjusted to be greater than three for the same reason. To complement the original trigger *Diphenhydramine Administration*, the following other drugs commonly used to manage hypersensitivity reactions in the studied ICUs were also included: dexchlorpheniramine, hydroxyzine, loratadine, promethazine, hydrocortisone, methylprednisolone, and prednisone. The *Anti-Emetic Administration* trigger was modified to include the three antiemetic medications used in the studied ICUs: ondansetron, bromopride, and metoclopramide. In the care module, of the 15 original triggers, only three were used: *Decrease in Hemoglobin or Hematocrit of 25% or Greater*, *Patient Fall*, and *Skin Rash* (included under the *Other* trigger). This selection was made because these triggers may be associated with ADRs, whereas the others are related to general adverse events not associated with medications. Additionally, a new module called *Laboratory Test Results* was introduced, containing the following triggers: *Sodium Less than 135 mEq/L*, *Potassium Less than 3.0 mmol/L*, *Potassium Greater than 5.5 mmol/L*, and *Platelets Less than 50,000*. This module was added because certain medications can alter laboratory parameters. Notably, this module is not part of the original GTT.

At the end of the adaptations, 28 triggers were utilized in this study, categorized as follows: i) medication module: PTT >50 s, INR >3, blood glucose <50 mg/dL, rising BUN or serum creatinine two times (2x) over baseline, ondansetron, bromopride, metoclopramide, phytomenadione, flumazenil, naloxone, protamine, dexchlorpheniramine, hydroxyzine, diphenhydramine, loratadine, hydrocortisone, methylprednisolone, promethazine, prednisone, over-sedation/hypotension, abrupt medication stop; ii) care module: decrease in hemoglobin or hematocrit of 25% or greater, patient fall, skin rash; iii) laboratory test results: sodium <135 mEq/L, potassium <3.0 mmol/L, potassium >5.5 mmol/L, platelets <50,000.

### 2.5 Triggers performance

Evaluating the performance of triggers involves analyzing factors such as the frequency of trigger occurrence in medical records ((1) Triggers per 100 medical records = number of times the trigger was identified in medical records/total number of medical records x 100), the frequency of ADRs identified by a trigger ((2) ADRs per 100 medical records = number of ADRs identified by a trigger/total number of medical records x 100), and the relative performance of each trigger in detecting an ADR (expressed as a percentage, %), defined by its positive predictive value (PPV), which is calculated by dividing the value obtained from (2) by the result from (1) and multiplying by 100.

### 2.6 Characterization of ADRs

ADRs were classified according to severity, using the WHO classification, as mild, moderate, or severe. The mechanism was classified using the Rawlins and Thompson classification (Rawlins and Thompson, 1977), which categorizes ADRs as type A or B. Causality was assessed using the algorithm proposed by Naranjo et al. (Naranjo et al., 1981) and the WHO classification (World Health Organization, 2013).

The analysis was conducted by two researchers–the author of this study and another pharmacist from the research group–both of whom had extensive experience with GTT. The results obtained were compared, and in cases of divergence, a third researcher, who was also experienced with the tool, provided the final characterization.

### 2.7 Classification of drugs suspected of causing ADRs

Drugs suspected of causing ADRs were classified according to the Anatomical Therapeutic Chemical (ATC) system, as designated by the WHO (World Health Organization, 2025).

### 2.8 ADRs notified by spontaneous reporting

The 2020 spontaneous reporting database at the HES was analyzed, and ADRs from the ICUs were identified.

### 2.9 Statistical analysis

The sample size was calculated using a pilot study to determine a statistically representative sample. The pilot study employed the adapted GTT to detect ADRs, assessing 52 patients chosen randomly from the 234 patients admitted to the G-ICU and C19-ICU.

Based on statistical calculations and considering that 71% of patients in the pilot study experienced at least one ADR, the total study sample size was 135 patients. A total of 135 patients admitted to the ICU during the study period were selected by lot, resulting in 76 (56.3%) patients in the G-ICU and 59 (43.7%) patients in the C19-ICU.

To describe the sample profile according to the study variables, frequency tables were created for categorical variables, with absolute frequency (n) and percentage (%) values, and descriptive measures (mean and standard deviation) for quantitative variables.

Comparisons between groups were made using the Chi-square or Fisher’s exact test for categorical variables and the Mann-Whitney U test for quantitative variables. Factors associated with the occurrence of ADRs (one or more) were identified using multiple logistic regression with stepwise selection criteria. The significance level adopted for the study was set to 5%.

Statistical analyses were conducted using The SAS System for Windows (Statistical Analysis System, version 9.4, SAS Institute Inc, 2002-2008, Cary, NC, United States).

## 3 Results

### 3.1 Study population

A total of 135 patients were included in the study, most of which were men (54.8%), with an average age of 61 ± 15.1 years and a length of hospital stay of 13.0 ± 11.0 days. The mortality rate in the ICU was 69.6%. When we separately compared the two ICUs included in the study, we observed that patients in the C19-ICU were admitted with greater severity than those in the G-ICU (higher SOFA scores at admission). For the other studied parameters, no differences were observed between the units (Table 1).

**TABLE 1:** Clinical and demographic data of the patients included in the study (n = 135).

Variables	G-ICU (n = 76)	C19-ICU (n = 59)	Total (n = 135)	p-value
Male sex, n (%)	39 (52.7)	35 (47.3)	74 (100.0)	0.3540^a^
Age, years (mean ± SD)	60.5 ± 15.6	62.3 ± 14.5	61.0 ± 15.1	0.5609^b^
Length of stay, days (mean ± SD)	12.2 ± 10.9	13.8 ± 11.0	13.0 ± 11.0	0.1438^b^
Death, n (%)	50 (53.2)	44 (46.8)	94 (100.0)	0.5439^a^
SOFA				
Admission SOFA (mean ± SD)	7.3 ± 4.5	8.9 ± 4.6	8.0 ± 4.6	**0.0450** ^b^
Minimum SOFA (mean ± SD)	4.7 ± 4.1	5.2 ± 4.1	4.9 ± 4.1	0.4862^b^
Maximum SOFA (mean ± SD)	11.3 ± 4.0	12.6 ± 4.1	11.9 ± 4.1	0.0520^b^
Average SOFA (mean ± SD)	7.9 ± 3.7	9.0 ± 3.8	8.4 ± 3.8	0.1090^b^

C19-ICU, COVID-19 Adult Intensive Care Unit; G-ICU, General Adult Intensive Care Unit; SD, sample standard deviation; SOFA, Sequential Organ Failure Assessment. Bold values = p < 0.05.

^a^ based on Chi-square test.

^b^ based on Mann-Whitney test.

At least one ADR was observed in 55 patients (40.7%). The C19-ICU had a higher number of patients with ADR (31; 52.5%) than patients without ADR (28; 47.5%) (Table 2). The total number of observed ADRs was 85, of which 65 (76.5%) were identified using the proposed triggers. No differences were observed among the units studied (Table 3).

**TABLE 2:** Adverse drug reactions identified in patients, according to the intensive care unit.

Variable	G-ICU	C19-ICU	Total	p-value
Patients with at least one identified ADR (n, (%))				
No	52 (68.4)	28 (47.5)	80 (59.3)	0.1147^a^
Yes	24 (31.6)	31 (52.5)	55 (40.7)	0.0501^a^
Total of patients (n, (%))	76 (100.0)	59 (100.0)	135 (100.0)	

ADR, adverse drug reaction; C19-ICU, COVID-19 adult intensive care unit; G-ICU, general adult intensive care unit; n, absolute number of patients.

^a^ based on the Chi-squared test.

**TABLE 3:** Number of adverse drug reactions identified with or without triggers in the study, according to the intensive care unit.

Variable	G-ICU	C19-ICU	Total	p-value
ADR identified by trigger (n (%))				
Yes	33 (50.8)	32 (49.2)	65 (100.0)	0.4631^a^
No	6 (30.0)	14 (70.0)	20 (100.0)	0.1919^a^
Total ADRs (n (%))	39 (45.9)	46 (54.1)	85 (100.0)	0.1344^a^

ADR, adverse drug reaction; C19-ICU, COVID-19 adult intensive care unit; G-ICU, general adult intensive care unit; n, absolute number of adverse drug reactions.

^a^ based on the Chi-squared test.

Age, sex, and length of hospitalization were related to the number of patients who presented or did not present with ADRs in the two studied ICUs (Table 4). Hospitalization days were associated with the presence of ADR in the two studied ICUs. Age was an important factor in the occurrence of ADR in the C19-ICU but not the G-ICU (Table 4).

**TABLE 4:** Association between age, sex, and length of hospitalization and adverse drug reactions in patients, according to the intensive care unit.

Patients				
Variables	without ADR	with ADR	Total	p-value
General adult intensive care unit				
	n = 52	n = 24	n = 76	
Age, years (mean ± SD)	59.6 ± 15.7	62.7 ± 15.3	60.5 ± 15.6	0.5203^a^
Hospitalization days (mean ± SD)	9.6 ± 8.4	17.8 ± 13.7	12.2 ± 10.9	**0.0020** ^a^
Male (n, (%))	29 (55.8)	10 (41.7)	39 (51.3)	0.2529^b^
Female (n, (%))	23 (44.2)	14 (58.3)	37 (48.7)	
COVID-19 adult intensive care unit				
	n = 28	n = 31	n = 59	
Age, years (mean ± SD)	56.4 ± 16.0	67.6 ± 10.7	62.3 ± 14.5	**0.0046** ^a^
Hospitalization days (mean ± SD)	9.1 ± 7.8	18.0 ± 11.8	13.8 ± 10.9	**<0.0001** ^a^
Male (n, (%))	13 (46.4)	22 (71.0)	35 (59.3)	0.0554^b^
Female (n, (%))	15 (53.6)	9 (29.0)	24 (40.7)	

ADR, adverse drug reaction; n, absolute number of patients; SD, sample standard deviation. Bold values = p < 0.05.

^a^ based on Mann-Whitney test.

^b^ based on the Chi-squared test.

Patients admitted to the C19-ICU had a 2.4 times higher risk of developing ADRs (Table 5). Patients with longer hospital stays had a 10.0% higher chance of developing an ADR. Although statistically significant, the increase in risk with age is small (3.9%) (Table 5).

**TABLE 5:** Multiple logistic regression for the presence or absence of adverse drug reactions, using the stepwise selection criterion.

Variables	OR (CI 95%)	p-value
Age	1.039 (1.010; 1.070)	**0.0085**
Hospitalization days	1.100 (1.050; 1.152)	**<0.0001**
C19-ICU	2.379 (1.082; 5.232)	**0.0311**

C19-ICU, COVID-19 adult intensive care unit; CI, confidence interval; OR, odds ratio. Bold values = p < 0.05.

**FIGURE 1:**
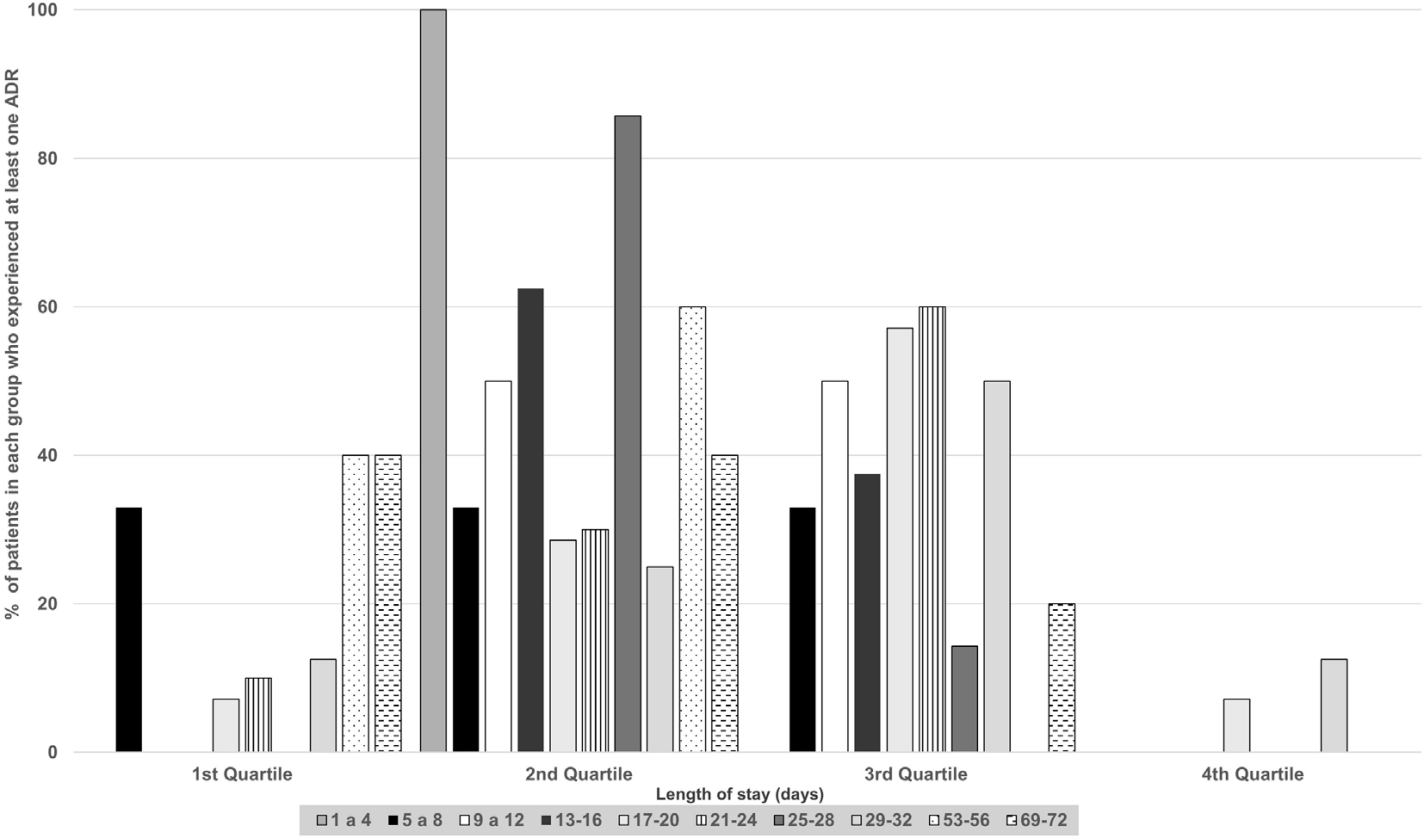
Temporal distribution of adverse drug reactions by quartiles of length of stay for patients from intensive care units.

An important aspect to evaluate is the timing of ADR occurrence during hospitalization. Figure 1 illustrates the temporal distribution of ADRs. Patients were categorized based on their length of stay, and the timing of ADRs was divided into quartiles. Regardless of the length of stay, Figure 1 indicates that most patients experienced ADRs during the second and third quartiles of hospitalization. For instance, 50.0% of patients who remained in the ICU for 9–12 days encountered ADRs in the second quartile, around the 8th day. Similarly, 60.0% of patients with a stay of 53–56 days experienced ADRs in the second quartile, approximately on the 28th day.

### 3.2 Triggers performance

All medical records in the study contained at least one trigger, totaling 1,182 triggers, with an average of 8.7 triggers per medical record. Notably, the presence of a trigger does not necessarily imply an ADR. Table 6 provides the results derived from the performance of all triggers employed in both ICUs.

**TABLE 6:** Characterization and performance of triggers used in the intensive care units.

Triggers	Trigger/100 medical records	ADR/100 medical records	PPV
PTT > 50	2.2	2.2	100.0
Skin Rash	1.5	1.5	100.0
Protamine	0.7	0.7	100.0
Hydroxyzine	0.7	0.7	100.0
INR > 3	6.7	3.7	55.6
Abrupt medication stop	49.6	22.2	44.8
Blood glucose <50 mg/dL	64.4	17.0	26.4
Platelets <50.000	15.6	3.7	23.8
Decrease in Hb/Ht of 25% or Greater	48.9	5.2	10.6
Ondansetron	25.9	2.2	8.6
Rising BUN or Serum Cr 2x over Baseline	58.5	2.2	3.8
Potassium <3.0 mmol/L	51.1	1.5	2.9
Over-Sedation/Hypotension (MAP <70)	112.6	3.0	2.6
Hydrocortisone	72.6	1.5	2.0
Bromopride	43.7	0.7	1.7
Metoclopramide	71.9	0.7	1.0
Potassium >5.5 mmol/L	96.3	0.7	0.8
Sodium <135 mEq/L	105.9	0.7	0.7
Methylprednisolone	23.0	0.0	0.0
Phytomenadione	8.9	0.0	0.0
Prednisone	7.4	0.0	0.0
Diphenhydramine	5.2	0.0	0.0
Promethazine	2.2	0.0	0.0
Naloxone	0.0	0.0	0.0
Flumazenil	0.0	0.0	0.0
Dexchlorpheniramine	0.0	0.0	0.0
Loratadine	0.0	0.0	0.0
Patient fall	0.0	0.0	0.0

ADR, adverse drug reaction; BUN, blood urea nitrogen; Cr, serum creatinine level; Hb, hemoglobin; Ht, hematocrit; INR, international normalized ratio; MAP, mean arterial pressure; PPV, positive predictive value; PTT, partial thromboplastin time.

The most frequently occurring triggers in the medical records, represented in the “Trigger/100 medical records” column, were over-sedation/hypotension (MAP <70) (112.6/100 medical records), sodium <135 (mEq/L) (105.9/100 medical records), and potassium (>5.5 mmol/L) (96.3/100 medical records). The triggers that most often identified an ADR, as shown in the “ADR/100 medical records” column, were abrupt medication discontinuation (22.2/100 medical records), blood glucose <50 mg/dL (17.0/100 medical records), and decrease in Hb/Ht of 25% or greater (5.2/100 medical records). Among the triggers used, those with the highest PPV, indicating their relative efficiency in identifying an ADR, were PTT >50, skin rash, protamine, and hydroxyzine, all with a PPV of 100%. In these cases, the presence of these triggers consistently indicated ADR occurrence.

### 3.3 Characterization of ADRs

When ADRs were classified according to severity, mechanism, and causality, no differences were observed between the two studied ICUs (Table 7). The ADRs identified in both the G-ICU and C19-ICU were predominantly moderate in severity and exhibited a type A mechanism classification, indicating that they are the most common, dose-dependent, and result from the increased pharmacological effects of the drug. In terms of causality, Naranjo’s algorithm indicated that the majority of ADRs in both ICUs were classified as possible, similar to the WHO causality classification.

**TABLE 7:** Characterization of the severity, mechanism, and causality of adverse drug reactions across the intensive care units studied.

Variables	G-ICU (n = 39)	C19-ICU (n = 46)	Total (n = 85)	p-value
Severity (n, (%))				
Mild	8 (20.5)	4 (8.7)	12 (14.1)	0.3148^a^
Moderate	21 (53.9)	27 (58.7)	48 (56.5)	
Severe	10 (25.6)	15 (32.6)	25 (29.4)	
Mechanism (n, (%))				
A	38 (97.4)	44 (95.7)	82 (96.5)	1.0000^a^
B	1 (2.6)	2 (4.3)	3 (3.5)	
Causality (n, (%))				
Naranjo Causality (n, (%))				
Possible	26 (66.6)	29 (63.0)	55 (64.7)	0.8047^b^
Probable	12 (30.8)	17 (37.0)	29 (34.1)	
Doubtful	1 (2.6)	0 (0.0)	1 (1.2)	
WHO Causality (n, (%))				
Inaccessible/Unclassifiable	0 (0.0)	0 (0.0)	0 (0.0)	—
Conditional/Unclassified	0 (0.0)	1 (2.1)	1 (1.2)	
Unlikely	0 (0.0)	1 (2.1)	1 (1.2)	
Possible	26 (66.6)	27 (58.7)	53 (62.3)	
Probable	13 (33.4)	17 (37.0)	30 (35.3)	
Certain/Defined	0 (0.0)	0 (0.0)	0 (0.0)	

C19-ICU, COVID-19 adult intensive care unit; G-ICU, general adult intensive care unit; n, absolute number of ADRs; WHO, World Health Organization.

^a^ based on Fisher’s exact test.

^b^ based on the Chi-square test.

### 3.4 Classification of drugs suspected of causing ADRs

Table 8 lists the drugs suspected of causing ADRs, detailing the number of ADRs attributed to each drug, along with their corresponding ATC classifications. The drugs most frequently associated with the occurrence of ADRs were insulin (an antidiabetic drug, including insulin and rapid-acting analogs), morphine (an opioid analgesic), and antithrombotic agents in the heparin group, such as heparin and enoxaparin (Table 8).

**TABLE 8:** Classification of drugs suspected in the occurrence of adverse reactions according to the Anatomical Therapeutic Chemical System, including the number and types of identified adverse drug reactions.

Medication	Number of ADRs identified	ADR type	Code	ATC classification
Regular insulin	22	Hypoglycemia	A10AB01	Antidiabetics (insulin and rapid-acting analogs)
Morphine	14	Constipation (13 times)/Skin Rash (1 time)	N02AA01	Analgesics (opioids)
Enoxaparin	7	Oral cavity bleeding (6 times)/Hematuria (1 time)	B01AB05	Antithrombotic agent (heparin group)
Heparin	7	Oral cavity bleeding (6 times)/Epistaxis (1 time)	B01AB01	Antithrombotic agent (heparin group)
Fentanyl	6	Bradycardia (4 times)/Chest rigidity (2 times)	N01AH01	Opioid anesthetic
Amikacin	3	Worsening renal function	J01GB06	Antimicrobial for systemic use (Other Aminoglycosides)
Amiodarone	2	Bradycardia	C01BD01	Class III antiarrhythmics
Bromopride	2	Diarrhea	A03FA04	Drugs for gastrointestinal disorders (Prokinetics)
Furosemide	2	Hypokalemia	C03CA01	Diuretics (loop diuretics)
RIPE	2	Vomiting	J04AM06	Antimycobacterial (combination of drugs to treat tuberculosis)
Risperidone	2	Decreased level of consciousness	N05AX08	Antipsychotics (other antipsychotics)
Tramadol	2	Nausea	N02AX02	Analgesic (other opioids)
Ampicillin + Sulbactam	1	Skin rash	J01CR01	Antimicrobial for systemic use (Penicillins with betalactamase inhibitor)
Amphotericin B	1	Hypokalemia	J02AA01	Systemic antimycotics (Antibiotics)
Carvedilol	1	Bradycardia	C07AG02	Beta-blocking agent
Clopidogrel	1	Bleeding in tracheostomy	B01AC04	Platelet aggregation inhibitors (except heparin)
Scopolamine	1	Paralytic ileus	A04AD01	Antiemetics and antinauseants
Spironolactone	1	Hyperkalemia	C03DA01	Diuretics (aldosterone antagonist)
Hydralazine	1	Hypotension	C02DB01	Antihypertensives (Smooth muscle agents)
Hydrochlorothiazide	1	Syndrome of inappropriate antidiuretic hormone secretion	C03AA03	Diuretics (Thiazides)
Lactulose	1	Diarrhea	A06AD11	Osmotic laxatives
Levomepromazine	1	Decreased level of consciousness	N05AA02	Antipsychotics
Metoclopramide	1	Psychomotor agitation	A03FA01	Drugs for gastrointestinal disorders (Prokinetics)
Neostigmine	1	Diarrhea	N07AA01	Parasympathomimetic (anticholinesterase)
Sulfamethoxazole+ Trimethoprim	1	Hyperkalemia	J01EE01	Systemic antimicrobials (Sulfonamides and Trimethoprim)
Warfarin	1	Upper digestive hemorrhage	B01AA03	Antithrombotic agent (vitamin K antagonist)

ADR, adverse drug reaction; ATC, anatomical therapeutic chemical; RIPE, association of rifampicin, isoniazid, pyrazinamide, and ethambutol.

### 3.5 ADRs notified by spontaneous reporting

Analysis of the spontaneous reporting database from 2020 revealed only five ADRs occurring in the ICU (all from the G-ICU and dermatological in nature, such as skin rashes and pruritus) were reported spontaneously. Analysis of these 5 ADRs revealed they were not from patients selected for randomization in the study. Therefore, none of the ADRs observed in the study were reported to the hospital, resulting in a 100.0% underreporting rate.

## 4 Discussion

Regarding the demographic characteristics of ICU patients, there was a slight predominance of males. A study published in 2011 (Vezzani et al., 2011) showed a male predominance among the total number of inpatients, with males comprising 64.0% of all patients admitted to adult ICU. Similarly, Marques et al. (Marques et al., 2020) showed that 55.8% of critically ill patients were male. Concerning the average age, one study analyzing 156 critically ill patients reported an average age of 54.9 years (Brito and Guirardello, 2012). Another study of critically ill patients found an average age of 51.2 years, ranging from 15 to 85 years (Eulálio et al., 2016). Regarding the average length of stay for critically ill patients, a study conducted in Rio de Janeiro in 2011 (Roque et al., 2016) reported an average of 8.9 days. However, our results showed a slightly higher average age and length of hospitalization than those in the aforementioned studies.

Several studies have used the SOFA score as a predictor of mortality in ICUs (Lambden et al., 2019; Raith et al., 2017; Polok et al., 2023). The SOFA score was initially developed to describe the morbidity of populations in various hospitalization environments such as ICUs. Today, after several validations, it is used as a determining factor in the diagnosis of sepsis (Lambden et al., 2019). Small variations in SOFA scores have been associated with changes in patient mortality rates. For instance, an increase in the SOFA score upon ICU admission is related to an increased mortality rate among these patients (Raith et al., 2017; Polok et al., 2023). In this study, critically ill patients had a mean SOFA score of approximately eight points throughout hospitalization, with a mean maximum SOFA score of approximately 12 points, and a mortality rate of approximately 70%. This aligns with the results of previous studies, which indicated that a higher SOFA score correlates with increased patient mortality. It is also consistent with the study by Anami et al. (2010), in which patients with a mean SOFA score of 6.01–7 had a mortality rate exceeding 50%, while those with a mean SOFA score above 10 had a 98.6% mortality rate.

In this study, less than half of the patients (40.7%) experienced at least one ADR. The proportion of ADRs found in medical records varies significantly across studies owing to various factors, including the methodologies and tools used and the number of researchers involved. A 2013 study conducted in a tertiary hospital with 128 patients reported that 15.6% of patients experienced at least one ADR during hospitalization (Rozenfeld et al., 2013), while other studies have indicated an ADR prevalence ranging from 2% to 24% (Franklin et al., 2010; Classen et al., 1991; Nilsson et al., 2012).

This study showed an association between length of hospitalization, age, and occurrence of ADRs. Aging and the concomitant use of multiple medications by elderly patients increase the likelihood of ADR occurrence (Rodrigues and de Oliveira, 2016). Furthermore, studies have indicated an association between the occurrence of ADEs, such as ADRs, and longer hospitalization (Roque and Melo, 2011; Classen et al., 1997). This study found that most patients in the G-ICU and C19-ICU experienced ADRs between the second and third quartiles of their hospitalization time; most ADRs were detected approximately halfway through the hospitalization period for each patient. Although no other studies have reported this association, this finding is important for directing pharmacovigilance actions and intensifying monitoring during periods of hospitalization that are most likely to result in ADR.

Patients admitted to the C19-ICU were found to be 2.4 times more likely to develop an ADR. Several factors may explain this, including the severity of COVID-19, which can lead to multiorgan dysfunction, such as in the liver and kidneys. These organs are crucial for drug metabolism and excretion, and their dysfunction can result in drug accumulation in the body, thereby increasing the likelihood of ADRs (Melo et al., 2021; Gérard et al., 2020).

Among the triggers used in this study, those with the highest PPV were PTT >50, skin rash, protamine, and hydroxyzine, all at 100%. This implies that each time these triggers appear, they identify an ADR. However, this result should not be relied upon solely because of the PPV, as protamine and hydroxyzine only appeared once in the medical records, and each identified a single ADR. Therefore, caution is advised when claiming that triggers with 100% PPV are the best for detecting ADRs. For example, blood glucose <50 mg/dL, despite having a PPV of 26.4%, appeared more frequently in medical records and was associated with a greater number of ADRs. Similarly, the “abrupt medication stop” trigger, with a PPV of 44.8% and a frequency of 49.6/100 medical records, identified 22.2 ADRs/100 medical records. Notably, more frequent triggers do not always identify ADR and are not necessarily associated with higher PPV. For instance, sodium <135 mEq/L (105.9/100 records) and potassium >5.5 mmol/L (96.3/100 records) have PPV of 0.7 and 0.8, respectively.

The results indicated that, in both ICUs, most ADRs were moderate, Type A, and classified as possible. These findings differ from those of previously published studies due to methodological differences. For instance, a 2020 study on critical patients in an emergency department reported that, according to the WHO classification, the majority of ADRs were classified as probable (Pandya et al., 2020). However, the results were similar regarding the mechanism, with most patients presenting Type A ADRs according to the Rawlins and Thompson classification, and regarding severity, with most patients presenting moderate ADRs according to the modified Hartwig and Seigel severity scale (Pandya et al., 2020). Another study, involving patients admitted to the ICU, found that most ADRs were mild according to a severity algorithm score, and the causality was classified as probable according to the WHO classification (Joshua et al., 2009).

Regular insulin, morphine, heparin, and enoxaparin were the drugs most frequently suspected to cause ADRs. The drug classes most frequently associated with ADRs in ICU environments include analgesics, sedatives, insulins, and anticoagulants (Esfahani et al., 2016). Hypoglycemia resulting from insulin use was the most commonly identified ADR in the ICUs studied. Cases of glycemic variation in critically ill patients are well documented in the literature (Kwan et al., 2020; Wang et al., 2021; Kwan et al., 2022). Hermanides et al. (Hermanides et al., 2010) reported that among the 5,961 patients evaluated, 4.6% experienced at least one episode of hypoglycemia, with insulin being the primary drug suspected to cause this ADR. Furthermore, hypoglycemia associated with insulin use has been linked to increased mortality in these patients. Hypoglycemia is the primary ADR associated with the continuous infusion of regular insulin owing to various factors such as excess insulin administration, hormonal deficiencies, concomitant medications, non-physiological conditions such as intubation, and variations in nutritional support (Almagthali et al., 2024).

Bleeding was also a frequent ADR observed in this study. It is common in ICUs, resulting from the use of anticoagulants and antiplatelet drugs, as well as invasive procedures such as tracheostomy and intubation, as reported in previous studies (Jonsson et al., 2021; Hariri et al., 2024). Additionally, cases of excessive drowsiness and delirium are notable, likely because of the concomitant use of central nervous system (CNS) depressants commonly used in ICUs (Wong et al., 2020). Opioids administered via infusion pumps for analgesia and sedation, combined with other CNS depressants, can lead to delirium and reduced consciousness associated with hypotension (Wu et al., 2023).

Finally, the use of triggers is essential for detecting ADRs that are not reported through spontaneous reporting. This technique has been proven to be highly effective in identifying ADRs, as it allows for the detection of a significantly higher number of ADRs and ADEs than spontaneous reporting methods (Classen et al., 2011). However, implementing this method can be challenging for institutions because it requires trained professionals with dedicated time for pharmacovigilance activities (Martins et al., 2018). The underreporting rate identified in this study was 100.0%, which aligns with the rate reported in a review of 37 studies by Hazell et al. (Hazell and Shakir, 2006). The high workload of the staff during the pandemic, as they prioritized patient care to ensure survival, was the primary factor contributing to this underreporting.

This study has several limitations. Evaluating physical medical records is time consuming, especially when assessing the presence of triggers. Laboratory test requests are generally described only in online medical records, whereas results are often found only in physical records. The retrospective nature of the study also posed a limitation, as it was not possible to discuss the 2020 cases with the G-ICU and C19-ICU staff owing to various factors, such as the high workload of health professionals. Consequently, the analysis relied solely on descriptions in medical records and nursing notes, which were sometimes handwritten. Another limitation is that only one researcher assessed the medical records, as no additional staff at the HES were available owing to the high workload, which may have affected the evaluation results of both physical and electronic records. Additionally, the influence of baseline clinical conditions on hospitalization duration was not analyzed, which is a critical omission, as these conditions may confound the interpretation of ADR prevalence. Finally, the possibility that some of the events observed in this study were medication errors and not ADRs cannot be ruled out.

## 5 Conclusion

The patients evaluated in this study had a mean age of approximately 61 years, were mostly male, spent an average of 13 days in hospital, and had a high SOFA score, which could be directly correlated with the high mortality rate observed. This study also revealed a high prevalence of ADRs in ICU patients. ADRs were associated with age and length of hospitalization. Additionally, patients admitted to the C19-ICU were 2.4 times more likely to develop an ADR.

Triggers such as “PTT >50” and “skin rash” proved to be effective in detecting ADRs, with a PPV of 100%. Most ADRs were moderate, Type A, and had possible causality. The most common ADRs were hypoglycemia and bleeding, which are often associated with the use of regular insulin and antithrombotic agents.

ADRs were identified more frequently using the adapted GTT, highlighting a significant under-reporting rate within the institution. Underreporting represents a major challenge in detecting ADRs, indicating the need to implement more effective active surveillance methods, such as trigger tools, in institutions. In conclusion, the use of triggers is essential for improving the detection of ADRs, significantly contributing to patient safety and the effectiveness of pharmacovigilance actions.

## Data Availability

The raw data supporting the conclusion of this article will be made available by the authors, without undue reservation.
